# Penetrating Injury to the Lower Limb by a Needlefish: A Case Report

**DOI:** 10.7759/cureus.107128

**Published:** 2026-04-15

**Authors:** Sabir Kumar Khadka, Khadheeja Abdulla, Nay Aung Zin, Mohamed Shareef Hussain, Mohamed Arsham Abdulrasheed

**Affiliations:** 1 Orthopaedics and Traumatology, Kulhudhuffushi Regional Hospital, Kulhudhuffushi, MDV

**Keywords:** belonidae, case report, emergency management, foreign body, lower limb injury, marine animal injury, needlefish injury, orthopedic trauma, penetrating trauma, soft tissue injury

## Abstract

Needlefish are elongated marine fish known for their high-speed leaps toward light sources, occasionally resulting in penetrating injuries in humans. We report a case of a 42-year-old fisherman who presented with a penetrating injury to the left leg caused by a needlefish beak. Clinical examination revealed a small entry wound with a protruding foreign body, and imaging confirmed multiple retained fragments within the soft tissue. The patient underwent surgical exploration and removal of the foreign bodies under spinal anesthesia, followed by wound debridement and irrigation. Due to the risk of contamination, the wound was left open to heal by secondary intention. The patient was managed with antibiotics, analgesics, and tetanus prophylaxis and recovered without complications. This case highlights the importance of early recognition, careful evaluation, and meticulous surgical planning in managing unusual marine-related penetrating injuries to prevent complications and ensure favorable outcomes.

## Introduction

Penetrating injuries to the extremities are commonly encountered in clinical practice, most often resulting from road traffic accidents, occupational trauma, or interpersonal violence [1]. However, injuries caused by marine animals are relatively rare and may present unique diagnostic and management challenges due to their mechanism and risk of contamination [2].

Needlefish (family Belonidae) are elongated, predatory marine fish characterized by sharp, beak-like jaws [3]. Although generally non-aggressive, they are known to leap out of the water at high speed, particularly when attracted to artificial light sources. These sudden, high-velocity movements can result in accidental injuries to individuals in close proximity, such as fishermen and divers.

Injuries caused by needlefish are uncommon but potentially serious, as the narrow, rigid beak can penetrate deeply into soft tissues and even vital structures, often causing more extensive internal damage than suggested by the external wound [4,5]. Additionally, the aquatic environment introduces a higher risk of infection, making prompt and appropriate management essential [6].

Due to the rarity of such injuries, there is limited awareness among clinicians, which may lead to delayed diagnosis or suboptimal management. Appropriate imaging plays an important role in identifying retained foreign bodies and guiding management [7].

This case report aims to highlight the clinical presentation, diagnostic approach, and surgical management of a penetrating lower limb injury caused by a needlefish, emphasizing the importance of early intervention and careful operative planning.

## Case presentation

**Figure 1:**
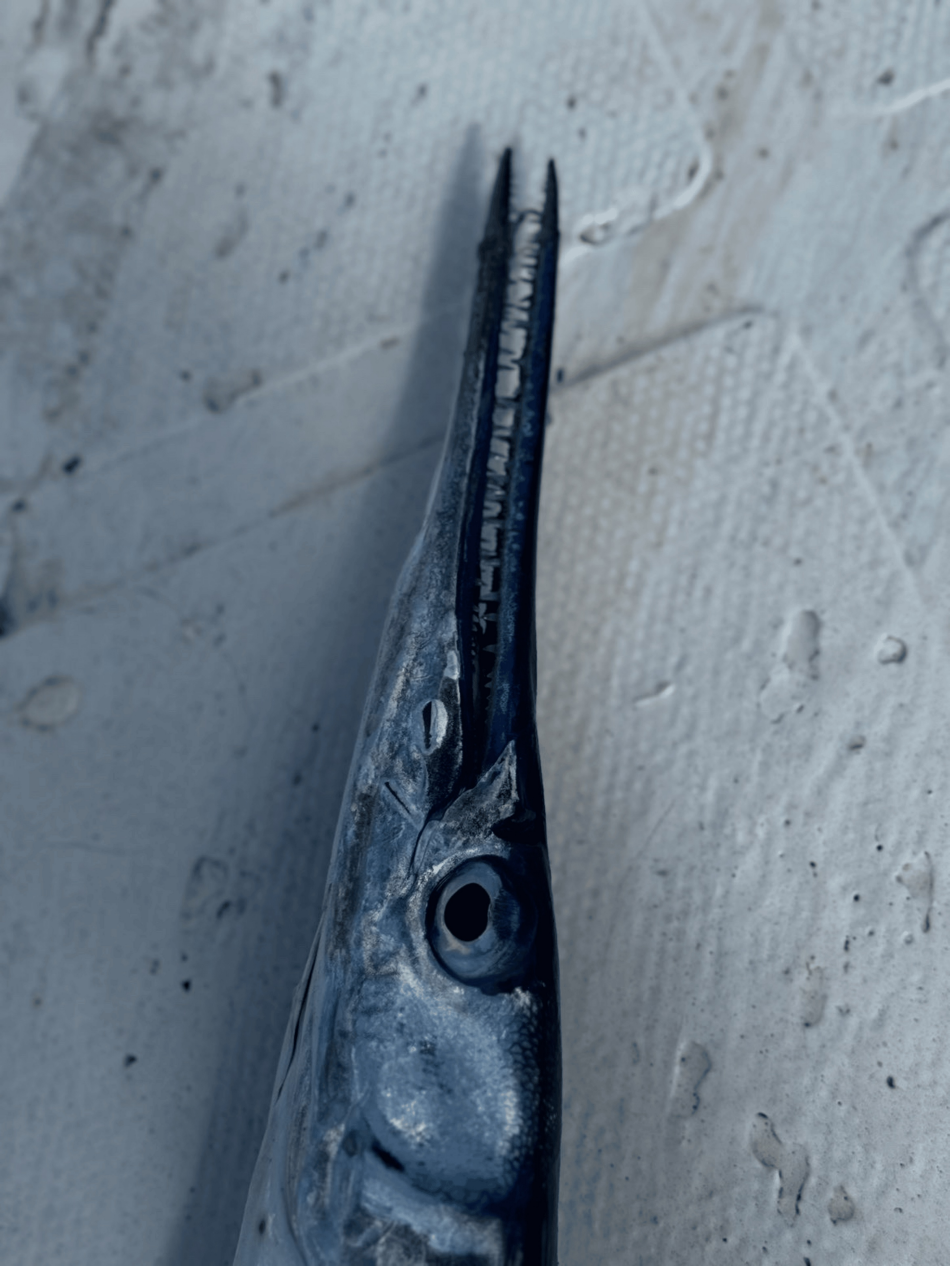
Needlefish specimen (original image by the author). Image demonstrating the elongated jaws and sharp beak capable of causing penetrating injuries.

A 42-year-old male fisherman presented to the emergency department four hours after sustaining a penetrating injury to his left leg while at sea. The patient reported that a needlefish, a marine fish characterized by an elongated, sharp beak (Figure 1), had leapt out of the water and struck his leg.

**Figure 2:**
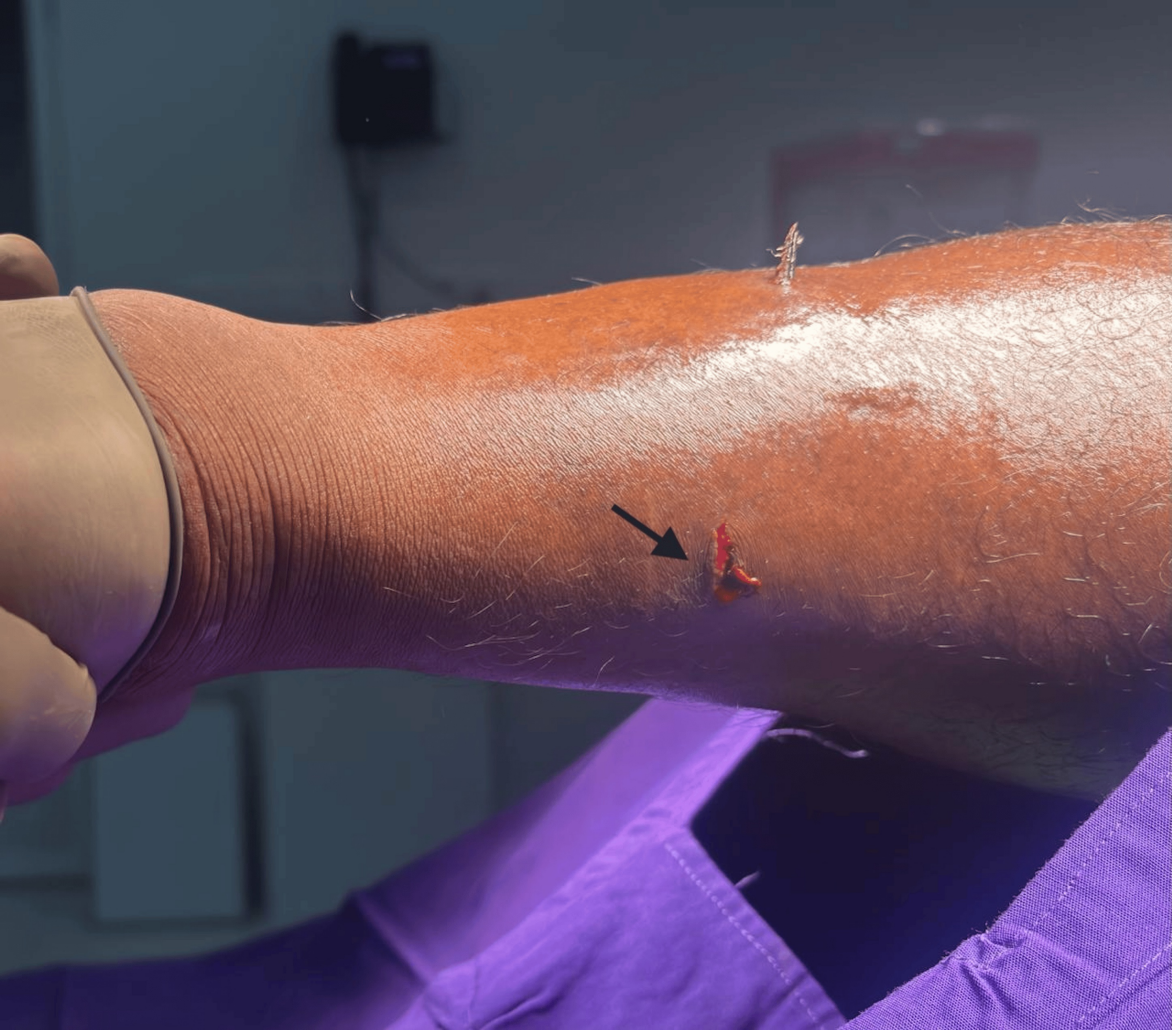
Clinical photograph of the left leg. The entry wound (black arrow) with a protruding foreign body fragment on the opposite side of the leg.

On clinical examination, a small but distinct entry wound was noted over the anterior aspect of the left leg, with a sharp foreign body fragment visibly protruding from the skin (Figure 2).

There was minimal external bleeding, with associated localized swelling and tenderness. Distal neurovascular examination was normal, with intact distal pulses and preserved motor and sensory function.

**Figure 3:**
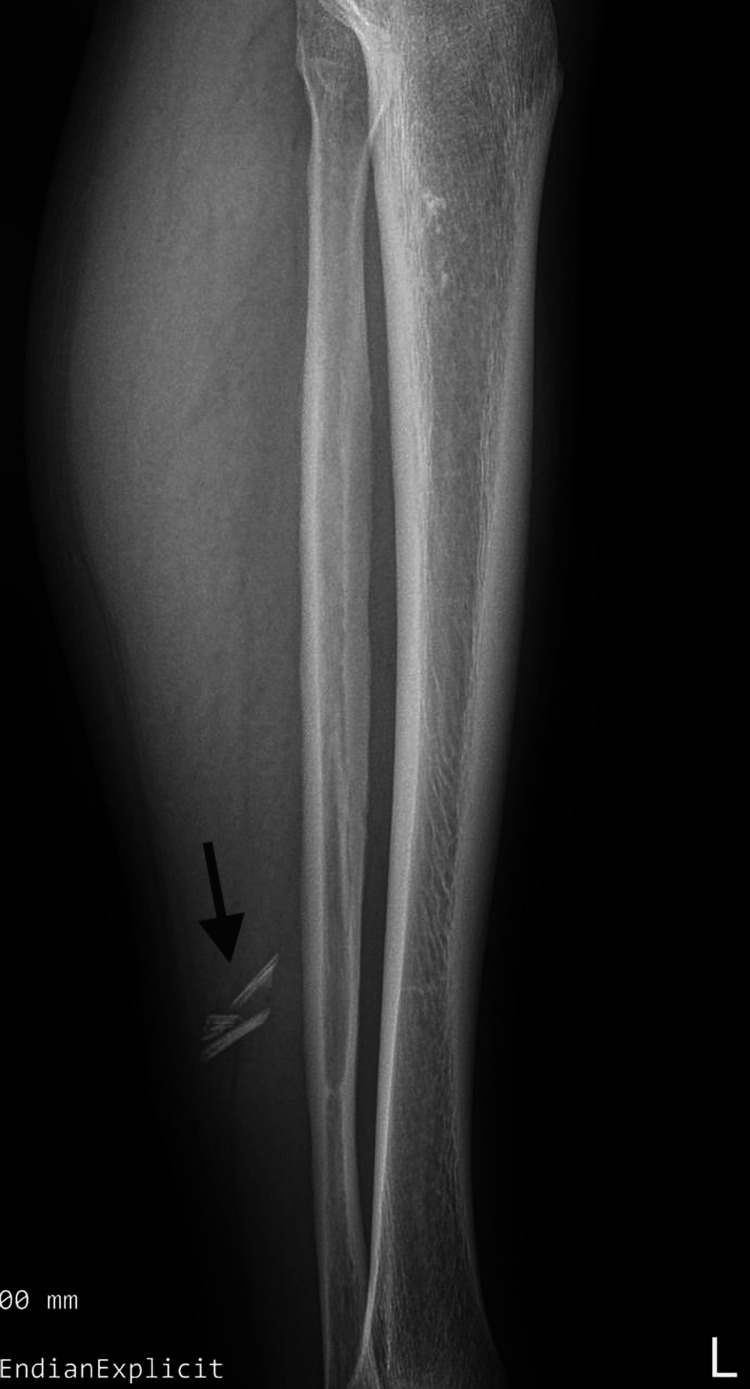
Plain radiograph of the left leg demonstrating multiple linear radiopaque foreign bodies (black arrow) within the soft tissue.

Plain radiography of the left leg revealed multiple linear radiopaque foreign bodies within the soft tissue, consistent with retained fragments of the needlefish beak (Figure 3).

**Figure 4:**
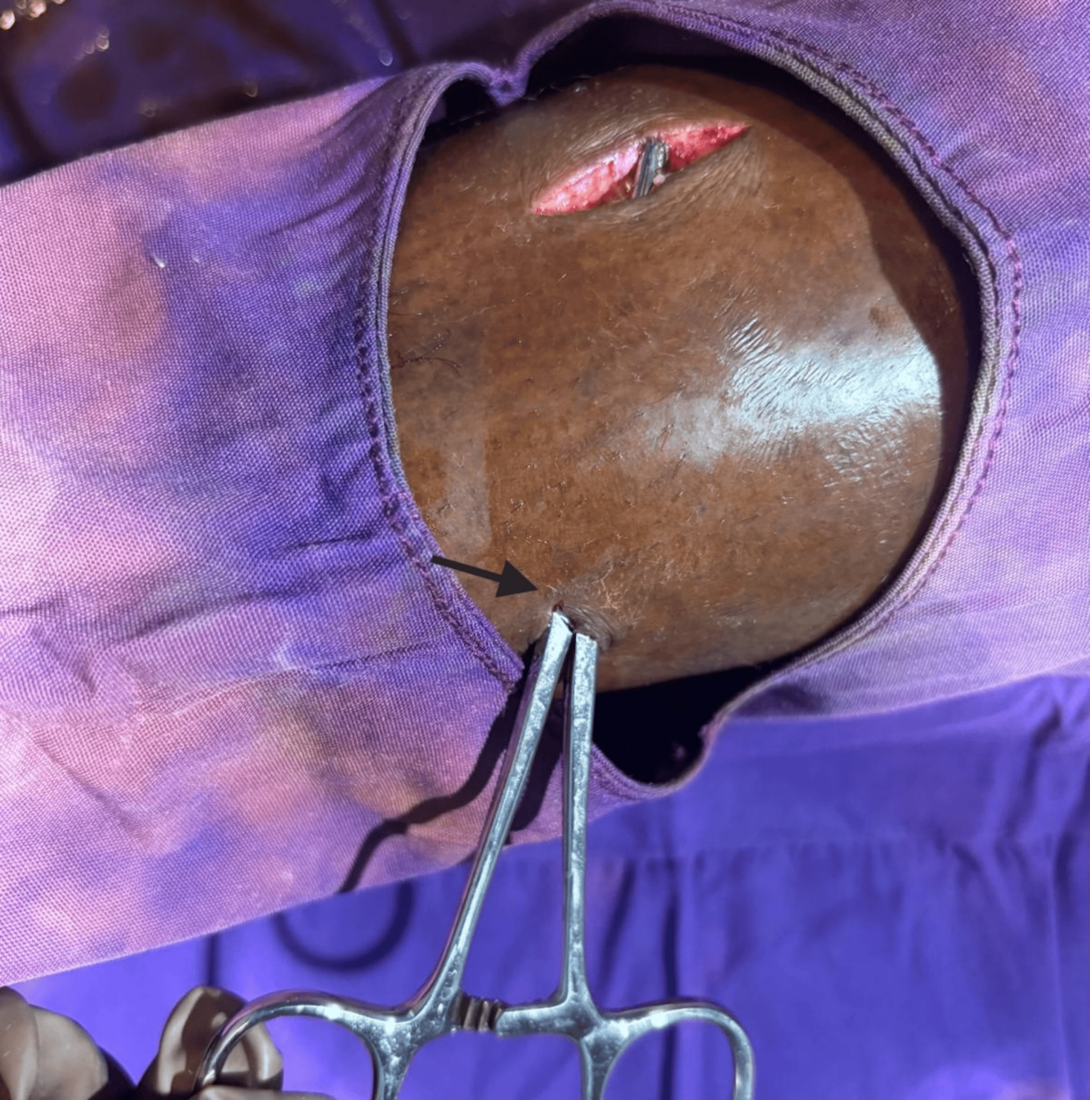
Intraoperative image demonstrating the sinus tract (black arrow) along the path of penetration.

The patient was taken to the operating theater for surgical exploration and removal of the foreign bodies under spinal anesthesia. After appropriate aseptic preparation and draping, the entry wound was carefully extended to allow adequate exposure. Gentle dissection was performed along the tract of penetration to avoid injury to surrounding soft tissues and neurovascular structures. A well-defined sinus tract was identified along the path of the foreign body (Figure 4).

**Figure 5:**
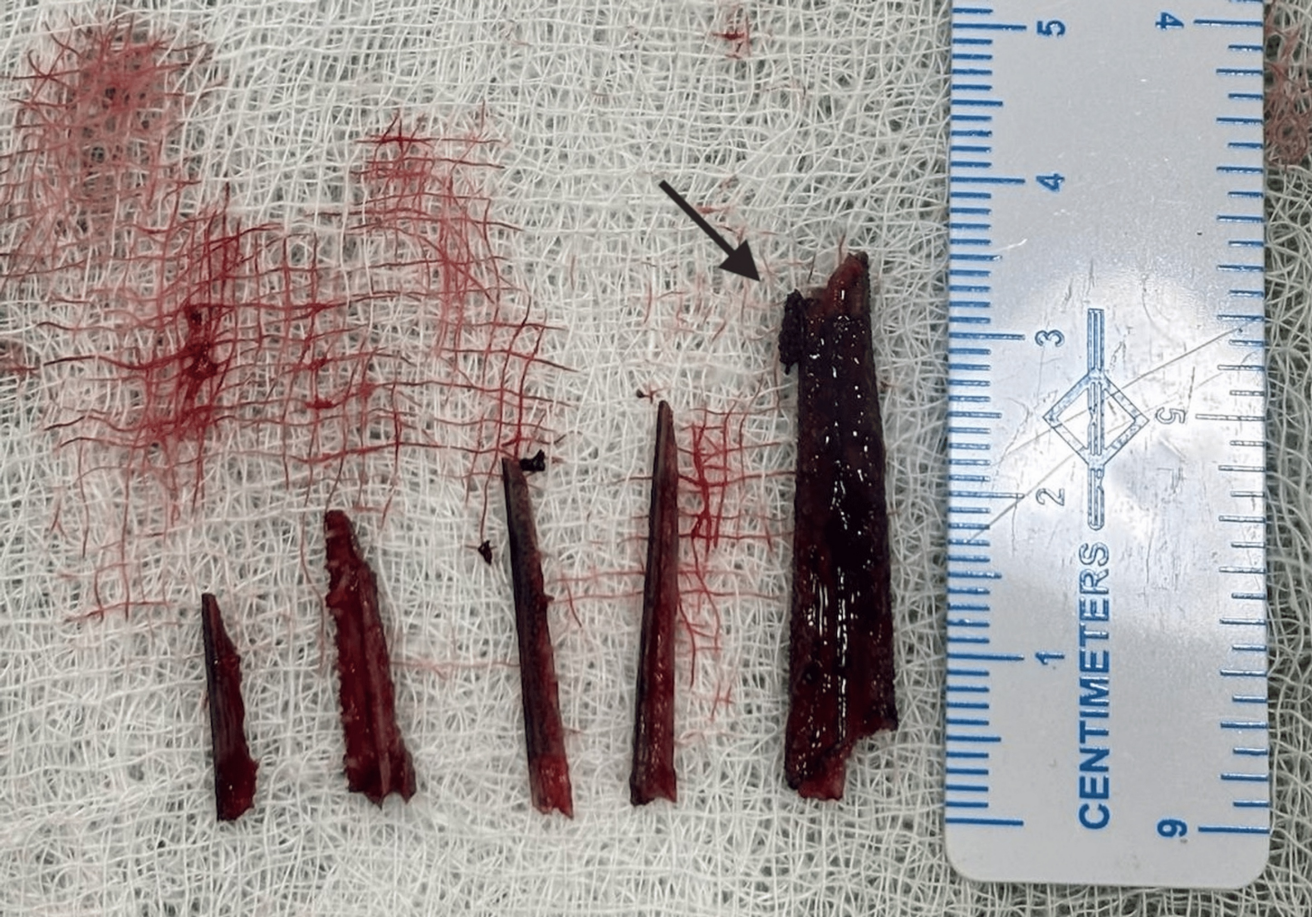
Extracted foreign body fragments. The black arrow indicates the largest fragment measuring approximately 3.5 × 1 cm.

Exploration revealed multiple elongated fragments of the needlefish beak embedded within the soft tissue. These fragments were meticulously extracted using surgical forceps, ensuring complete removal of all foreign material. The largest fragment measured approximately 3.5 × 1 cm (Figure 5).

Copious irrigation with normal saline was performed, followed by thorough debridement. The wound was left open to heal by secondary intention.

Postoperatively, the patient was managed with intravenous antibiotics, analgesics, and tetanus prophylaxis. The recovery was uneventful, and the patient demonstrated satisfactory wound healing with no complications on follow-up.

## Discussion

Penetrating injuries to the extremities are commonly encountered in clinical practice, most frequently resulting from road traffic accidents, occupational hazards, or interpersonal trauma [1]. However, injuries caused by marine animals are relatively rare and often present unique diagnostic and therapeutic challenges due to their mechanism and environmental contamination [2].

Needlefish-related injuries, although uncommon, have been reported in various anatomical regions, including the orbit and spinal cord, demonstrating their potential to cause significant morbidity [4,5]. Unlike typical penetrating trauma, these injuries occur due to high-velocity leaps of the fish, and the elongated, rigid beak enables deep tissue penetration. As a result, the extent of internal injury may be disproportionately greater than the external wound, necessitating a high index of suspicion during assessment.

A key aspect of management in such cases is the risk of infection associated with aquatic injuries. Marine environments harbor a wide range of pathogenic organisms, and penetrating injuries sustained in these settings are particularly prone to contamination. Therefore, early administration of appropriate broad-spectrum antibiotics is essential to reduce the risk of complications such as cellulitis, abscess formation, and delayed wound healing [6].

Careful clinical evaluation and surgical planning are critical prior to the removal of any foreign body. Blind or unplanned extraction may result in further injury to surrounding soft tissues and neurovascular structures. A thorough history, including the mechanism of injury and identification of the causative organism, plays an important role in guiding management.

Imaging plays a crucial role in the assessment of retained foreign bodies. Plain radiography is often the first-line and most accessible modality for detecting radiopaque objects such as fish beaks [7]. However, clinicians should be aware of its limitations, particularly in identifying radiolucent materials or objects located adjacent to bony structures, in which case advanced imaging such as computed tomography may be warranted.

Comparison with previously reported cases suggests that outcomes are generally favorable when early recognition and appropriate surgical management are achieved [4,5]. Conversely, delayed presentation, incomplete removal of foreign body fragments, or inadequate wound care may lead to complications such as persistent inflammation, infection, and retained foreign body reactions [6,7].

In the present case, prompt presentation, accurate history-taking, and meticulous surgical exploration under spinal anesthesia allowed complete removal of all foreign body fragments without complications. The absence of neurovascular injury and appropriate postoperative management contributed to a favorable clinical outcome.

## Conclusions

Needlefish-related injuries are rare but can result in penetrating trauma with retained foreign body fragments. This case highlights the importance of careful clinical assessment, appropriate imaging, and planned surgical exploration for complete removal of foreign material. The use of wound debridement and leaving the wound open for secondary healing helped reduce the risk of infection in this contaminated injury. Early and appropriate management in this case resulted in a favorable outcome without complications.
